# Aqueous Dispersion of Manganese–Zinc Ferrite Nanoparticles Protected by PEG as a T_2_ MRI Temperature Contrast Agent

**DOI:** 10.3390/ijms242216458

**Published:** 2023-11-17

**Authors:** Dorota Lachowicz, Angelika Kmita, Marta Gajewska, Elżbieta Trynkiewicz, Marek Przybylski, Stephen E. Russek, Karl F. Stupic, David A. Woodrum, Krzysztof R. Gorny, Zbigniew J. Celinski, Janusz H. Hankiewicz

**Affiliations:** 1 Academic Centre for Materials and Nanotechnology, AGH University of Krakow, 30-059 Krakow, Poland; dbielska@agh.edu.pl (D.L.); marta.gajewska@agh.edu.pl (M.G.); elzbieta.trynkiewicz@agh.edu.pl (E.T.); marprzyb@agh.edu.pl (M.P.); 2 Faculty of Physics and Applied Computer Science, AGH University of Krakow, 30-059 Krakow, Poland; 3 National Institute of Standards and Technology, 325 Broadway St, Boulder, CO 80305, USA; stephen.russek@nist.gov (S.E.R.);; 4 Department of Radiology, Mayo Clinic, 200 First Street SW, Rochester, MN 55905, USA; woodrum.david@mayo.edu (D.A.W.); gorny.krzysztof@mayo.edu (K.R.G.); 5 Center for the BioFrontiers Institute, University of Colorado Colorado Springs, 1420 Austin Bluffs Parkway, Colorado Springs, CO 80918, USA; zcelinsk@uccs.edu (Z.J.C.); jhankiew@uccs.edu (J.H.H.)

**Keywords:** MnZn ferrite, PEG coating, nuclear relaxation times, MRI thermometry, motion averaging regime, laser ablations

## Abstract

Mixed manganese–zinc ferrite nanoparticles coated with PEG were studied for their potential usefulness in MRI thermometry as temperature-sensitive contrast agents. Particles in the form of an 8.5 nm core coated with a 3.5 nm layer of PEG were fabricated using a newly developed, one-step method. The composition of Mn_0.48_Zn_0.46_Fe_2.06_O_4_ was found to have a strong thermal dependence of magnetization in the temperature range between 5 and 50 °C. Nanoparticles suspended in an agar gel mimicking animal tissue and showing non-significant impact on cell viability in the biological test were studied with NMR and MRI over the same temperature range. For the concentration of 0.017 mg/mL of Fe, the spin–spin relaxation time T_2_ increased from 3.1 to 8.3 ms, while longitudinal relaxation time T_1_ shows a moderate decrease from 149.0 to 125.1 ms. A temperature map of the phantom exposed to the radial temperature gradient obtained by heating it with an 808 nm laser was calculated from T_2_ weighted spin-echo differential MR images. Analysis of temperature maps yields thermal/spatial resolution of 3.2 °C at the distance of 2.9 mm. The experimental relaxation rate R_2_ data of water protons were compared with those obtained from calculations using a theoretical model incorporating the motion averaging regime.

## 1. Introduction

The well-known dependence of magnetization on temperature [1] has rarely been explored for the purpose of utilization of magnetic nanoparticles as a Magnetic Resonance Imaging (MRI) temperature-sensitive contrast agent [2,3]. This sensitivity may provide additional functionality to magnetic particles with potential applications in MRI-guided thermal ablations [4].

Our motivation in this project is to explore thermal sensitivity of magnetic particles to potentially improve the safety, reliability, and effectiveness of MRI-guided thermal therapies where real-time temperature monitoring is essential [4,5,6]. Conventional thermometry is usually invasive, allows only single-point temperature measurements, and may interfere with the MRI image formation [7]. The dependence of proton resonance frequency on temperature (0.01 ppm/°C) forms the basis of the so-called proton resonance frequency shift (PRF) thermometry, which is commonly used clinically to monitor temperature changes in tissues [8]. In practice, PRF often fails, reducing the use of ablation procedures. PRF can suffer from artifacts caused by tissue motion, field disturbances caused by systemic static magnetic field drift, changes in the magnetic susceptibility distribution, and the presence of adipose tissue [9].

We propose an MRI thermometry method utilizing the magnetic nanoparticles-based contrast agent that avoids issues inherent to PRF thermometry and produces fast, robust, spatial temperature maps superimposed on anatomical images within the targeted tissues. Previously, we have shown that large, micrometer-size, magnetic particles produce a local temperature-dependent magnetic field, leading to changes in the apparent transverse relaxation time, $T_{2}^{*}$. This results in hypointense regions in $T_{2}^{*}$-weighted gradient echo MR images [10,11]. As the temperature increases, the magnetization of the particles is reduced, and the signal intensity in the MRI image increases. The difference in image intensity due to temperature changes allows one to measure the temperature in a specific area.

As large magnetic particles cannot be administered intravenously, we are now exploring the possibility of using superparamagnetic particles made of mixed ferrites as an injectable temperature-sensitive contrast agent. The dry magnetic particles suitable for MRI thermometry must meet the following criteria: the Curie temperature (T_C_) is near body temperature (37.0 °C), the diameter must be around 10 nm, it must be soluble in water, and it must be non-toxic. Additionally, in a water solution, one needs a short T_1_ relaxation time to speed up the MRI acquisition. We recently proposed using mixed CuZn polyethylene glycol (PEG)-coated nanoparticles as a temperature-sensitive MRI contrast agent [12]. Unfortunately, the CuZn aqueous solutions possessed a rather long T_1_ relaxation time (above 600 ms), and temperature-dependent images required long acquisition times.

In this paper, we describe the results of the fabrication and characterization of mixed manganese–zinc ferrite nanoparticles. Because of the existence of a strong magnetic dipolar interaction, ferrite nanoparticles tend to agglomerate, thus preventing them from dissolving in water. To avoid agglomeration, MnZn ferrite nanoparticles were stabilized by the formation of a PEG layer on their surfaces during the one-step fabrication process described earlier [12]. PEG is a biocompatible polymer widely used to increase the nanoparticles’ dispersibility [13]. These coated nanoparticles, dispersed in tissue model hydrogels, produce temperature-dependent dipolar magnetic fields that change the nuclear spin–spin T_2_ relaxation of water protons. When T_2_ is strongly temperature dependent, the T_2_ weighted spin-echo MR provides images with intensities that are temperature dependent. The experiments were conducted in a realistic setting with a laser light diffuser used in clinical MRI-guided laser ablations, which produced a strong radial temperature gradient across the phantom [14].

We chose manganese ferrite as the base for the particle’s core due to its known high value of magnetization and relatively low Curie temperature among 3d metal ferrites [15]. Additionally, recent preclinical studies describe Mn-based chelated compounds as suitable for MRI intravascular contrast enhancement and neuronal connection studies [16,17,18,19]. We hypothesize that doping ferromagnetic manganese ferrites with diamagnetic zinc ions will modify their properties, making them useful for MRI thermometry. We expected that by increasing the Zn concentration, due to a reduction in the exchange interactions between tetrahedral and octahedral sites of the ferrite, the T_C_ will decrease even further [20,21]. We specifically focused on the composition of Mn_0.5_Zn_0.5_Fe_2_O_4_. For this composition, T_C_ decreases from 300 °C for the pure MnFe_2_O_4_ to temperature around 150 °C in a bulk material. In the nanoparticle form of MnZn ferrites, similar trends are observed though they are modified by surface effects [22,23].

## 2. Results

PEGyled magnetic nanoparticles (MNPs) were synthesized in a one-step synthesis (for details, see the Materials and Methods section) through the thermal decomposition of organometallic precursors in a polymer matrix (PEG). To obtain stable nanoparticles of desired compositions, the appropriate quantities of precursor salts (Mn(acac)_2_, Zn(acac)_2_, and Fe(acac)_3_) and PEG (1000) were mixed. Keeping in mind future applications, a low molecular weight of PEG 1000 was used to avoid PEG accumulation in the liver and lysosomes of normal tissues. The synthesized nanoparticles formed a highly colloidally stable aqueous solution. No particle aggregation or precipitation was observed over a period of 6 months.

### 2.1. Chemical and Morphological Characterization

**Figure 1:**
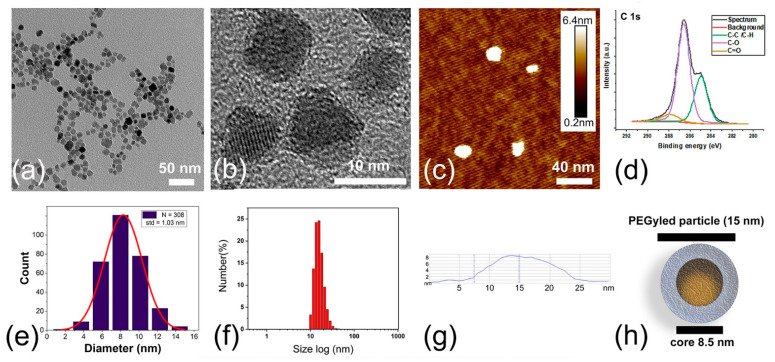
Morphological characterization of PEG-coated Mn _0.48_ Zn _0.46_ Fe _2.06_ O_4_ nanoparticles. (**a**) Representative TEM image. Note that the PEG coating of the core particles cannot be seen by TEM due to the low contrast between the polymer and carbon-coated grid. (**b**). High-resolution TEM image of nanoparticles. (**c**) Topographic image of nanoparticles deposited on Si plate as measured by AFM in air. (**d**) High-resolution XPS spectra of C1s for obtained nanoparticles. (**e**) The size distribution histogram of nanoparticles based on TEM data (red line—Gaussian distribution).(**f**) Distribution profiles of the hydrodynamic diameters (d_h_) obtained from DLS. (**g**) Nanoparticle’s cross-section profile (AFM). Measurement of the diameter of an isolated nanoparticle. (**h**) Cartoon of the proposed structure of a nanoparticle showing the nanocrystal core and polymer coating.

Based on the existing literature on doping iron oxide nanoparticles [24,25,26], the formation of a ferrite of formula Mn_x_Zn_y_Fe_2-x-y_O_4_ was expected. The target composition in our technological effort was Mn_0.5_Zn_0.5_Fe_2_O_4_ for reported values of high magnetization and lowest Curie temperature [27,28,29]. The final chemical composition of MNPs determined from the inductively coupled–plasma optical emission spectroscopy (ICP-OES) measurements was Mn_0.48_Zn_0.46_Fe_2.06_O_4_. The morphology and size distribution of the obtained objects were determined using transmission electron microscopy (TEM) (Figure 1a,b and Figure S3), atomic force microscopy (AFM) (Figure 1c,g) and dynamic light scattering (DLS) (Figure 1f and Figure S4) methods. The obtained MNPs exhibited an irregular sphere-like shape. Micrographs and a histogram are presented in Figure 1b and Figure 1e, respectively. EDX analysis confirmed the presence of iron, manganese and zinc in the obtained ferrite nanoparticles (Figure S1, Table S1). The obtained nanoparticles were core–shell structures (Figure 1h) with cores visible in the polymeric matrix.

The analysis of particles from the TEM image in Figure 1a,b gives particle core sizes at 8.5 ± 1.0 nm (mean and standard deviation). The hydrodynamic size of the entire particle (ferrite core and PEG shell) was measured with a DLS technique. The obtained value of 20 nm agrees with AFM measurements (15 nm), but as expected, it is bigger than the one obtained from TEM [30]. The small particle size with strong temperature-dependent magnetization is essential to achieve our goal of obtaining water-soluble temperature-sensitive particles suitable for MRI thermometry.

XPS analysis was performed to confirm the presence of a PEG layer in the structure of the received nanoparticles. The XPS survey spectrum of the MNPs revealed peaks at 1021, 710, 640, 530, and 285 eV corresponding to the Zn2p, Fe2p, Mn2p, O1s, and C1s lines, respectively (Figure S2). The C1s spectra of MNPs were curve fitted with three different peaks using a Shirley-type background subtraction (Figure 1d). The binding energies of 285, 286.6 and 288.9 eV can be attributed to the C–C/C–H, C–O, and O–C–O/C=O groups of the polyethylene glycol coating, respectively. The synthesis method used here has been modified to a one-step process in which nanocrystallites are formed and stabilized simultaneously. Thanks to this modification, the obtained nanoparticles exhibit long-lasting colloidal stabilization. The presented high-resolution XPS spectra for C1s confirm the presence of undegraded PEG polymer on the surface of the particles.

### 2.2. TG/DTG Analysis

**Figure 2:**
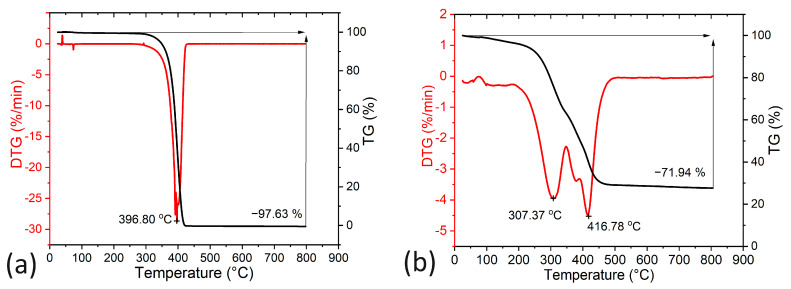
Comparison of TG/DTG curves at heating rate β = 10 °C × min^−1^ under argon atmosphere: (**a**) pure PEG 1000. (**b**) PEG 1000-coated MnZn nanoparticles.

The thermal decomposition of pure PEG 1000 and PEG-coated MnZn nanoparticles as shown in Figure 2 was investigated using thermogravimetric (TG) and differential thermogravimetric (DTG) analysis. In the case of the pure PEG sample (see Figure 2a), it was found that the beginning of one-stage thermal decomposition occurred at a temperature of 352 °C with the maximum weight loss rate at 396.8 °C. The residual weight of 2.37% at 800 °C is attributed to decomposition products [12,31,32].

However, in the case of the PEG-coated MnZn nanoparticles, the results indicate that the progressive thermal decomposition of PEG adsorbed on the surface of the MnZn nanoparticles occurred in two stages: at 307.37 °C and 416.78 °C (see Figure 2b). This is confirmed by the characteristic inflections on the TG and DTG curves. This effect can be attributed to the strong interfacial interactions of MnZn nanoparticles with the PEG 1000 polymer [32], as shown. The total weight loss of the sample was approximately 72%, which confirms that the surface of the MnZn nanoparticles was covered with PEG polymer. The PEG polymer undergoes thermal degradation. The results of the TG/DTG analysis were used to determine the mass of the ferrite core of PEG-coated MnZn nanoparticles in the magnetic properties studies presented below.

### 2.3. Magnetic Properties

**Figure 3:**
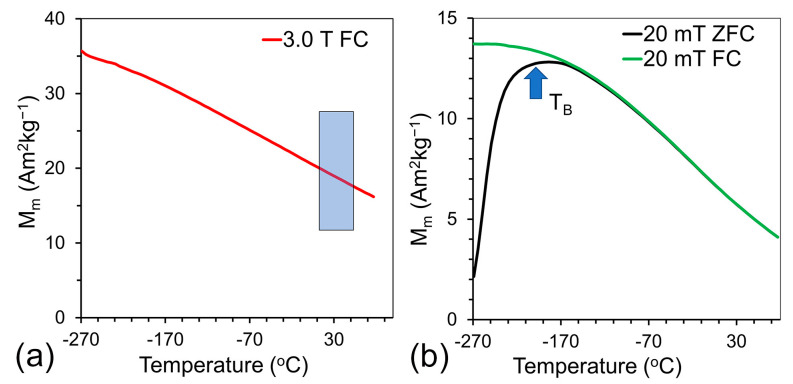
Magnetization measurements for dried PEG-protected Mn-Zn ferrite nanoparticles. (**a**) Results at 3.0 T field. Blue rectangle marks the range of 5 to 50 °C, where NMR and MRI experiments were conducted. (**b**) Results at 20 mT, zero field cooling (ZFC) and field cooling (FC). From these results, we estimate the blocking temperature (T_B_, marked with blue arrow) in the range of −198 °C.

Figure 3 shows magnetization measurements of nanoparticles in a dry form. One can appreciate the strong temperature dependence of the magnetization in 3.0 T magnetic fields (see Figure 3a). Note that 3.0 T is the field of an MRI scanner used for temperature-dependent studies. The slope value of 7.1 × 10^−2^ Am^2^kg^−1^ °C^−1^ is in the same range as the reported earlier values we obtained using micrometer-sized ferrite particles, which were fabricated by ceramic technology [11]. Zero field cooling (ZFC) and field cooling (FC) measurements were conducted at 20 mT magnetic field (see Figure 3b) to determine the blocking temperature, which is about −198 °C, indicating the superparamagnetic nature of nanoparticles. The estimated Curie temperature of particles is approximately 120 °C, which is similar to T_C_ values reported in the literature for bulk materials [33,34].

### 2.4. Nuclear Relaxation

**Figure 4:**
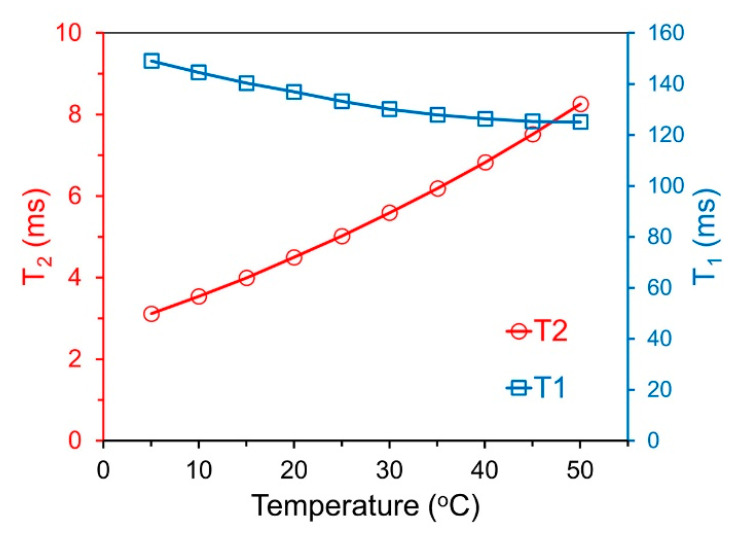
Temperature dependence of nuclear relaxation times of water protons in agar gel with embedded MnZn nanoparticles in concentration of 0.017 mg/mL of Fe at 3.0 T magnetic field. Red circles depict transverse T_2_ nuclear relaxation times, blue squares depict longitudinal T_1_ time. Note that longitudinal and transverse relaxation times have slopes with different signs.

Figure 4 presents the temperature dependence of transverse relaxation times T_2_ and longitudinal times T_1_ of water protons suspended in the concentration of 0.017 mg/mL in 1% *w*/*w* agar gel. T_2_ relaxation times show a very strong temperature dependence, increasing by 165% as the temperature changes from 5 to 50 °C. This steep temperature increase suggests that the intensity of T_2_-weighted MRI images will be a good indicator of temperature when using Mn-Zn nanoparticles in aqueous solutions. The strong temperature-dependent T_2_ allows for the possibility of using standard spin-echo MRI acquisition rather than previously used gradient echo methods for T_2_* weighting reported earlier [10]. Using spin-echo MRI would be advantageous because of the reduced susceptibility and chemical shift artifacts in spin-echo measurements [35,36]. In contrast, the longitudinal relaxation values of T_1_ shown in Figure 4 change only by 16%, which is much less than T_2_.

### 2.5. Magnetic Resonance Imaging

**Figure 5:**
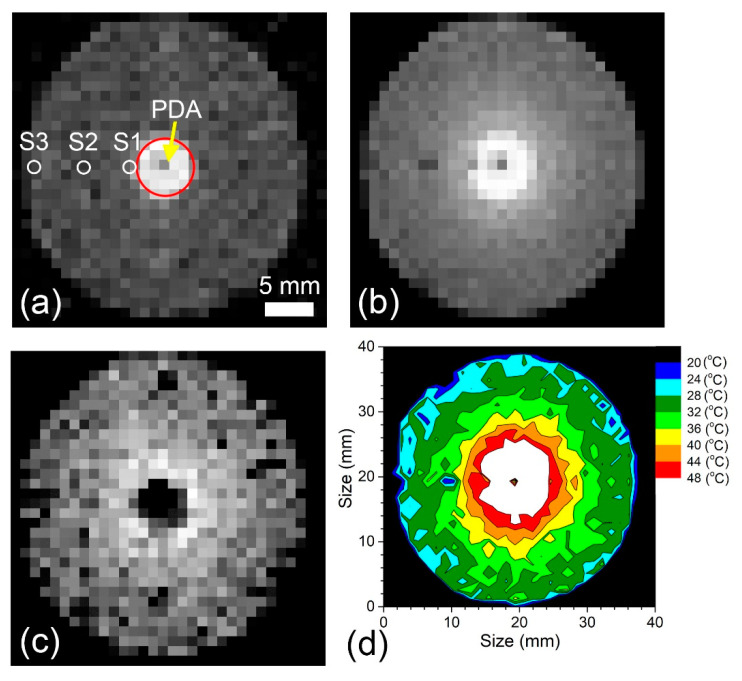
Spin echo T_2_-weighted MRI results in the presence of a strong radial temperature gradient produced by a laser diffuser at the center of the phantom made of 1% agar gel with embedded 8.5 nm Mn_0.48_Zn_0.46_Fe_2.06_O_4_ particles with a concentration of 0.017 mg/mL of Fe. (**a**) Image before heating. Small white circles show the position of MRI-compatible, miniature temperature sensors S1, S2 and S3. (**b**) Image after heating. (**c**) Difference image (**a**,**b**). (**d**) Pseudo-color temperature map with 4 °C isotherms. Black spot in the center shows the position of the laser diffuser.

To determine the spatial/thermal resolution of the method, we designed an MRI compatible setup. An 808 nm, 5 W continuous wave laser beam, sent through a glass fiber terminated with a light diffuser, produced an in-plane temperature gradient across the phantom (see the Materials and Methods section for design details). The cylindrical phantom is 30 mm across, 20 mm tall, and is made of 1% agar gel with embedded 8.5 nm Mn_0.48_Zn_0.46_ Fe_2.06_O_4_ particles with a concentration of 0.017 mg/mL of Fe. With this setup, we were able to produce a maximum 30 °C temperature difference across 14 mm of agar gel with embedded particles. Examples of axial T_2_-weighted MR images of the agar phantom with studied nanoparticles before and after heating are shown in Figure 5. Figure 5a is an axial image of the phantom before heating taken at a magnet’s bore temperature of 17.5 °C. Three miniature, MRI-compatible, optical sensors are marked with white circles and denoted as S1, S2 and S3. The white part of the image in the center, marked with a large red circle, delineates the agar gel filled with polydopamine (PDA) particles to aid heating as described in the Materials and Methods section. The PDA core is 6 mm in diameter and is excluded from further thermal analysis. An image of the phantom in the same location after 5-min laser heating is shown in Figure 5b.

Figure 5c shows the results of subtraction of image intensity obtained before heating (Figure 5a) from the image intensity obtained after heating (Figure 5b). Note the radial dependence in the brightness. Image difference data were used to obtain a pseudo-color temperature map with 4 °C isotherms, as exhibited in Figure 5d.

### 2.6. Diffusion Measurements

Apparent diffusion coefficient (ADC) of water molecules in deionized water (DIW) and in agar–DIW solutions results are given in Table 1. We note the strong temperature dependence of ADC and only a slight decrease in water molecules’ mobility with agar concentration. The presented results are in agreement with previously published data [37,38,39]. The error in the diffusivity measurements, as determined by comparison with consensus data for deionized water, is less than ±3%.

### 2.7. Biological Study

**Figure 6:**
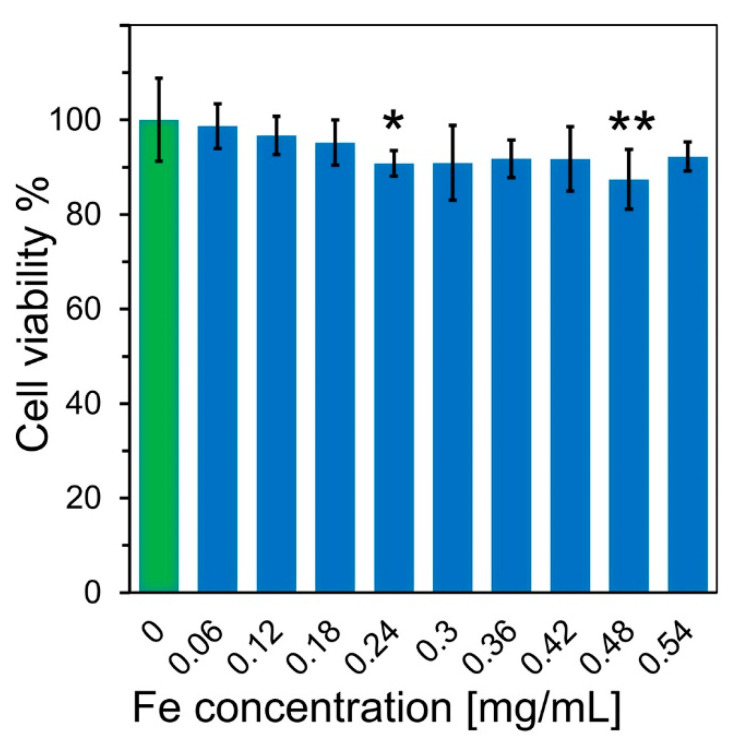
Results of the MTT assay conducted for murine neuroblastoma cells incubated for 24 h with MnZn nanoparticles at different concentration of Fe. Values are expressed as a percentage of the control, which was defined as a green bar (100%). Bars represent the mean and black error bars represent standard deviations of obtained data (*n* = 6). * *p* <0.05, ** *p* <0.01 compared with the control.

Considering the potential medical applications of nanoparticles, it is necessary to determine the particles’ cytotoxicity. We performed preliminary toxicity studies by examining the viability of murine fibroblast cells treated with a dispersion of obtained magnetic nanoparticles using the MTT assay for cellular metabolic activity [40]. As seen in Figure 6, the cell viability drops with the nanoparticle concentration. Statistical analysis conducted using ANOVA with a Dunnett’s comparison test shows that for concentrations above 0.24 mg/mL of Fe, the viability is significantly lower than in the control group (*p* < 0.05). Results indicate that MnZn nanoparticles used in a concentration of 0.017 mg/mL of Fe in these phantom studies should be safe for in vivo MRI studies. DNA damage results are still pending. In the future, with magnetic particles embedded in polymers, the issue of direct biocompatibility will be eliminated because some of the polymers are already approved for use by the US Food and Drug Administration [41].

## 3. Discussion

In this section, we discuss subjects relevant to the potential future application of nanoparticles as MRI contrast agents for MRI-guided laser ablations such as predictions of spin–spin relaxation rate ($R_{2}=\frac{1}{T_{2}}$) using a motion averaging regime model, the calculation of spatial–thermal resolution of temperature determination, a method of enhancement of the contrast using combined T_2_ and T_1_ weighting of the spin-echo MR images, and limitations of MRI thermometry using a temperature-sensitive contrast agent.

### 3.1. Spin–Spin Relaxation in Motional Averaging Regime

Reports on the nuclear relaxation of water protons due to the presence of magnetic nanoparticles of the size around 10 nm, both coated and not coated, suggest that the value of T_2_ is determined by the status of the motional regime of the water about the nanoparticles [42,43,44,45].

Following these reports, we assumed that for PEG-coated MnZn nanoparticles used in this project, the water molecule diffusion process averages the phase variation of proton spins originating from the local magnetic field inhomogeneities. In this situation, the NMR signal decay is mainly due to magnetic dipolar interactions between nuclear spins (protons) and unpaired electron spins on the nanoparticles [46]. Consequently, we hypothesized that our experiments were conducted in a motional averaging regime. In this regime, one expects that $∆\omega \tau_{D}<1$, where Δω is a Larmor frequency shift of water protons in the magnetic field created by a nanoparticle at its equator at the closest accessible distance, $\tau_{D}=\frac{d^{2}}{4D}$ is the diffusion time, *d* is the particle size and *D* is the diffusion coefficient [24]. The diffusion time can be interpreted as the time required for a water molecule to move in the distance of d, the average particle diameter, in any given direction. For the calculations, the shape of MnZn ferrite nanoparticles covered by a layer of PEG (impermeable coating) was approximated by the sphere of *d* = 15 nm diameter as shown schematically in Figure 1h.

**Figure 7:**
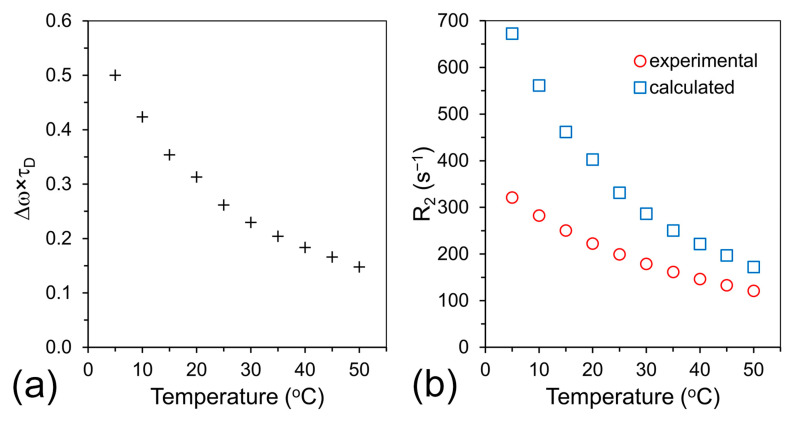
Temperature dependence of water molecules motion regime and water proton R_2_. (**a**) Values of $∆\omega \tau_{D}$. (**b**) Comparison of experimental (red circles) and calculated data of R_2_ (blue squares). Note that no fitting parameters were used for calculations of R_2_.

The numerical value of the frequency shift was determined from the equation: $∆\omega =\frac{1}{3}\gamma \mu_{o}M_{v}$ where γ is a gyromagnetic factor of protons (2.675 × 10^8^ rads^−1^T^−1^), $\mu_{o}$ is the magnetic permeability of vacuum ($\sim 4\pi 10^{-7}TmA^{-1}$) and $M_{v}$ is the volume magnetization calculated from the SQUID data presented in Figure 3a using the value of density of 5.0 gcm^−3^ obtained from bulk ferrite measurements [47]. Since magnetization and ADC are strongly temperature dependent, calculations were conducted for the entire temperature range from 5 to 50 °C. The temperature dependence of the product of characteristic frequency and translational diffusion time ($∆\omega \tau_{D}$) are presented in Figure 7a. The calculation indicates that at 5 °C, we are on the border line of the motional averaging regime and with a temperature increase, $∆\omega \tau_{D}$ asymptotically reaches a value of 0.1, which is well in the motion-averaging regime.

Using the known model of water molecules in the motion-averaging regime, we apply the following Equation (1) to calculate the spin–spin relaxation rate *R*_2_ at a high magnetic field [42,48]

(1) $$R_{2}=\frac{1}{T_{2}}=\frac{16}{45}f\tau_{D}{\left(\Delta \omega \right)}^{2}$$

where *f* is the volume fraction occupied by nanoparticles in the agar–water solution of 0.017 mg/mL of Fe.

A comparison of the results presented in Figure 7b shows a strong discrepancy between experimental and calculated values of R_2_ at low temperature (109%).

This discrepancy slowly vanishes at higher temperatures, and at 50 °C, the calculated values of R_2_ are about 42% higher than the experimental values. This improvement can be explained in terms of much higher water diffusion at higher temperatures (see Table 1 for diffusion values) and consequently a better-defined averaging regime at higher temperatures ($∆\omega \tau_{D}<0.15$) than at lower temperatures ($∆\omega \tau_{D}>0.5$). Nevertheless, Equation (1) gives us a good starting point for the prediction of R_2_ values in the temperature range from 36 to 60 °C during laser ablations experiments.

### 3.2. Spatial–Thermal Resolution of the Temperature Determination

**Figure 8:**
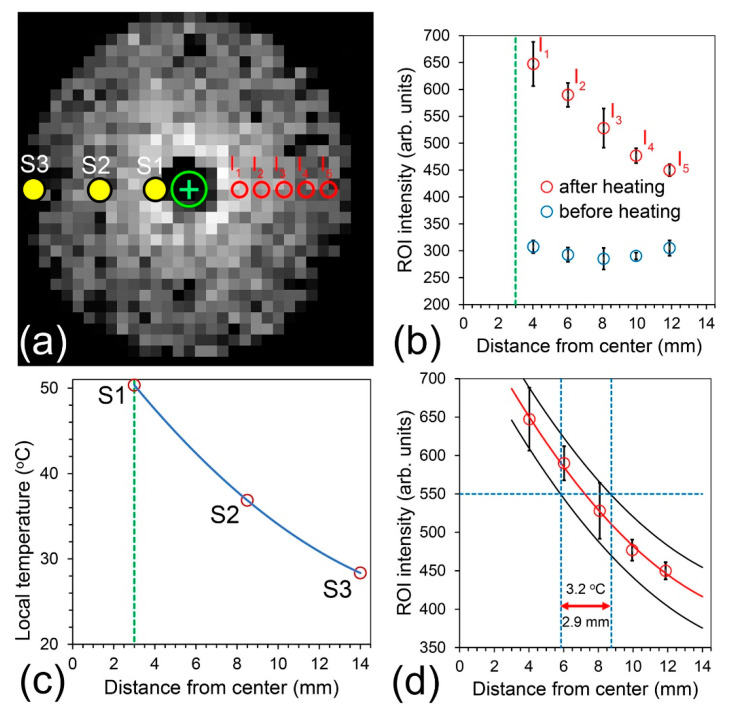
Determination of the in-plane thermal–spatial accuracy. (**a**) Location of temperature sensors S1, S2 and S3, and regions of interests (ROIs) I_1_ through I_5_ on the differential image. (**b**) Local brightens at ROI positions from N = 4 pixels (mean and standard deviation). Note that the green dashed line delineates the position of the PDA core that was excluded from analysis. (**c**) Local temperature at incorporated sensors (red circles). Blue solid line represents the fit of experimental data points to polynomial function. (**d**) Method of thermal/spatial resolution determination (3.2 °C at 2.9 mm) using 100% confidence bands (solid black lines). Data points are mean values and the corresponding standard deviations.

Figure 8 illustrates the analyses method of image intensity in terms of spatial–thermal accuracy. Figure 8a shows the location of five regions of interest (ROIs), I_1_ through I_5_, on the differential image. Each ROI covers N = 4 pixels. The mean values from each ROI and their standard deviations are presented in Figure 8b. Figure 8c shows the local temperature measured with three miniature MRI-compatible sensors in the middle of an MRI scan, as shown in Appendix A section. Data from these points were fitted to a second-order polynomial function (blue solid line: y = 0.08x^2^ − 3.4x + 59.9, R^2^ = 1) for the determination of temperature as a function of position. Due to the presence of sensors on the left hand of the image and pixel intensity disturbances in this area, we analyzed pixels on opposite side of the image.

Figure 8d shows the ROI data points (red circles) with their own polynomial fit (red solid line: y = 1.16x^2^ − 44.3x + 809, R^2^ = 0.997). Two confidence bands were created for 100% coverage of the standard deviations (black solid lines). The intermediate intensity of 550 (blue horizontal dashed line) was arbitrarily chosen for a determination of how accurately we can estimate the temperature in space. Intersection points with confidence bands were projected on the distance axis (blue dashed vertical lines). The obtained intersections were assigned to temperatures from the polynomial function obtained from a regression analysis of the three data points presented in Figure 8c. The results are 3.2 °C over 2.9 mm, meaning we can distinguish temperature changes of 3.2 °C at 2.9 mm distance.

### 3.3. Comparison of MnZn with CuZn

We reported the use of mixed CuZn ferrite nanoparticles as temperature-sensitive contrast agents for MRI previously [12]. Table 2 summarizes the main results obtained from the previous and current studies. The major difference between MnZn and CuZn nanoparticles was a much bigger mass magnetization at 40 °C of the former, 18.4 Am^2^kg^−1^ and 11.8 Am^2^kg^−1^, respectively. MnZn nanoparticles also have a much faster temperature drop of magnetization than CuZn, 14.4% and 7.3%, respectively. Despite the much smaller concentrations of MnZn nanoparticles used in this study (0.017 mg/mL vs. 0.128 mg/mL of Fe), their high magnetization immediately leads to higher values of corresponding relaxivities of water protons $r_{1,2}=\frac{R_{1,2}}{C}$, where *C* is the nanoparticles concentration in mM of Fe. The shorter T_1_ for protons in the presence of MnZn nanoparticles allows for much faster MRI data acquisition, which is necessary for a fast temporal resolution of temperature determination (16 s vs. 256 s).

As shown in Figure 4, in the Results section above, T_2_ and T_1_ exhibit monotonic temperature dependence with slopes of different signs. This “sheer-like” pattern of the thermal dependence of T_2_ and T_1_ was also visible in work on CuZn nanoparticles [12]. This observation provides an interesting avenue for improvement in temperature contrast that can be achieved by simultaneous T_2_ and T_1_ weighting, which is similar to the reported T_1_-T_2_ dual mode MRI [49,50]. In general, the temperature contrast enhancement in aqueous solutions of an exogenous agent resulting in temperature positive T_2_ and negative T_1_ slopes can be obtained using a single standard spin-echo sequence with optimized times of TE (responsible for T_2_ weighting) and of TR (responsible for T_1_ weighting). However, practical uses of this strategy require a much steeper temperature change in T_1_ and will be a subject of further study.

### 3.4. Limitations of Thermometry Using MRI Temperature-Sensitive Contrast Agent

The proposed method of temperature determination using temperature-dependent intensity of T_1_, T_2_ and $T_{2}^{*}$-weighted MR images suffers inherently from two major issues. As we have shown in our earlier work, the nuclear relaxation of water protons in the presence of magnetic particles strongly depends on magnetic field magnitude and particle concentration [51,52]. While the magnetic field of the MR scanner is well determined and does not change, the concentration of particles injected into the blood arteries will vary in space and time. This challenge can be partially mitigated by using particle-doped polymer filaments or patches and inserted into or placed on the region of interest, as proposed in our recent papers [51,53]. The calibration of the temperature-dependent image intensity of such ferro-polymers can be conducted prior to their use. We envision that one would obtain temperature distribution, 1D from the filament or 2D from the patch, being in thermal contact with the tissue, during the MRI-guided procedure.

## 4. Materials and Methods

### 4.1. Materials

Poly(ethylene glycol) (1000 Da, BioUltra), zinc(II) acetylacetonate (puriss. p.a., ≥95%, Sigma-Aldrich, Burlington, NJ, USA), iron(III) acetylacetonate (puriss. p.a., 99.9%, Sigma-Aldrich, Burlington, NJ, USA), manganese(II) acetylacetonate (puriss. p.a., ≥97.0%, Sigma-Aldrich, Burlington, NJ, USA), acetone (puriss. p.a., POCH S.A, Gliwice, Poland), and diethyl ether (puriss. p.a., Sigma Aldrich, Burlington, NJ, USA) were used as received. Millipore-quality water was used during the experiments.

### 4.2. Synthesis of PEG Coated Nanoparticles

**Figure 9:**
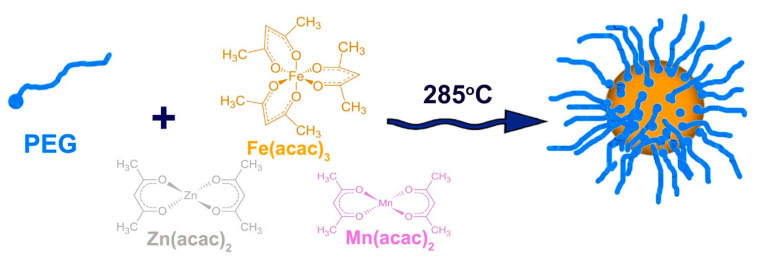
Graphic diagram of the fabrication of PEG-coated MnZn ferrite nanoparticles.

Magnetic nanoparticles (MNPs) were synthesized through the thermal decomposition of organometallic precursors in a polymer matrix (PEG) described in detail earlier [12]. Figure 9 illustrates the fabrication of the materials. Manganese (II) acetylacetonate, zinc (II) acetylacetonate and iron (III) acetylacetonate in the presence of poly(ethylene glycol) were used to obtain colloidal stable nanoparticles.

The first step was to heat 7 mmol PEG to 80 °C for 10 min under an argon atmosphere, stirring continuously on a magnetic stirrer. Then, 0.6 mmol of Fe(acac)_3_ and 0.2 Zn(acac)_2_, 0.2 Mn(acac)_2_ were added to the molten PEG and intensively stirred at 80 °C under argon for 30 min. Next, the solution was quickly heated to 285 °C and kept at this temperature for 60 min. The obtained mixture was cooled at a temperature of 60 °C, and then 20 mL of toluene was added. After cooling to room temperature, the mixture was washed with acetone and diethyl ether. This mixture was purified by magnetic separation. Organic solvents were disposed of and replaced with pure water, where the nanoparticles were finally suspended.

### 4.3. Morphology Characterization Methods

Transmission Electron Microscopy (Tecnai TF 20 X-TWIN (FEI)) was used to determine the morphology and size distribution of nanoparticles. TEM and high-resolution HR-TEM figures were analyzed using Image-J 1.54d software. The chemical composition was checked using Energy-Dispersive X-ray Spectroscopy (EDX). The size distribution and the zeta potential of the nanoparticles were measured with the Malvern Nano ZS apparatus (Malvern Instrument Ltd., Worcestershire, UK). The chemical composition of the obtained nanoparticles was analyzed with inductively coupled–plasma optical emission spectroscopy (ICP-OES) using the Thermo Scientific iCAP 7000 Plus apparatus. All samples were digested in a concentrated supra pure nitric acid. Further chemical characterization was performed using an X-ray photoelectron spectroscopy (XPS). Measurements were completed in a PHI 5000 VersaProbe II spectrometer with an Al Kα radiation source (E = 1486.6 eV). The working pressure in the analytical chamber was less than 3 × 10^−7^ Pa. High-resolution spectra were measured at the analyzer pass energy set to 49.95 eV. To compensate for the charge-up effect, we used a dual-beam charge neutralizer. All binding energies were corrected to a C-C line at 284.8 eV. The spectrum background was subtracted by the Shirley method. The PHI MultiPak software ver 9.3.0.3 was used for data analysis.

### 4.4. Thermogravimetric Analysis (TGA)

The thermal decomposition of the PEG-coated MnZn nanoparticles was evaluated by thermogravimetric analysis. The TG and DTG experiments were carried out using a Thermogravimetric Analyzer SDT Q600 (TA Instruments, New Castle, DE, USA). Analysis of samples of pure MnZn and PEG-coated MnZn ferrite nanoparticles was performed from room temperature to 800 °C with the constant heating rate β = 10 °C × min^−1^ under argon atmosphere. The thermogravimetric results were processed with Universal Analysis 2000 software [54].

### 4.5. Magnetization Measurements

The temperature dependence of dry nanoparticles magnetization was measured using a Superconducting Quantum Interference Device (SQUID) magnetometer in the temperature range from −269 to 77 °C (Quantum Design, San Diego, CA, USA). To determine their superparamagnetic behavior, particles were measured at a low field of 20 mT using a zero-field cooled and field cooled protocols. Additional measurements at 3.0 T were conducted with the sample temperature initially lowered to −269 °C in a field of 3.0 T that corresponds to the fields of MRI scanners used for imaging. The mass magnetization was calculated using the corrected mass value of the ferrite core from thermogravimetric measurements that show that the ferrite core constitutes only 28.1% of the total (shell and core) particle mass.

### 4.6. Nuclear Relaxation

It is essential for this project to accurately determine the thermal dependence of the water protons’ nuclear relaxation times T_1_ and T_2_. Temperature-dependent measurements were conducted at 3.0 T in the range from 5 to 50 °C with 5 °C steps. Relaxation times were measured using a conventional pulsed NMR spectrometer (NMR Redstone console; Tecmag, Houston, TX, USA and a 3.0 T, 54 mm superconducting magnet; Oxford Instruments, Abingdon, UK) [55]. The temperature of the sample was regulated and stabilized through the flow of gaseous nitrogen through the radio-frequency probe head containing a sample. The sample temperature uncertainty was determined to be ±0.2 °C. Ten minutes were allowed between setting the target temperature and the beginning of the NMR measurements to achieve the thermal equilibrium within the sample. Prior to the measurement at 5 °C, the magnet was shimmed using the automated Berger Brown sequence [56] to achieve homogeneity under 5 Hz. T_1_ relaxation was measured with the inversion recovery sequence with the following parameters: the inversion 180° pulse 26.8 μs, separated from the sampling 90° pulse of 13.4 μs by 20 variable delay time covering the range from 6 ms to 4.171 s and a recovery time of 2.5 s. Spin–spin T_2_ relaxation time was determined using a spin-echo Carr–Purcell–Meiboom–Gill sequence with the following parameters: excitation 90° pulse 13.4 μs, focusing 180° pulse = 26.8 μs, refocusing time 2.0 ms, 20 refocusing periods from 4 to 44 ms and a recovery time of 2.2 s.

### 4.7. Diffusion Measurements

The diffusion data of water molecules were obtained by imaging of 5 mm diameter NMR tubes filled with deionized water and with 0.5%, 1.0% and 2.0% *w*/*w* concentration of agar gel in DIW in a temperature-controlled phantom using a preclinical MRI operating at 3.0 T with a voxel size of 1 mm × 1 mm × 2 mm. The temperature was measured using a calibrated fiber optic thermometer with an accuracy of ±0.25 °C. The apparent diffusion coefficients were measured using a standard pulsed-gradient spin-echo sequence (PGSE) sequence using 5 b-values ranging from 0 to 1000 s/mm^2^ [57]. The following imaging geometry parameters for the diffusion encoded spin-echo multi-slice (SEMS) sequence were used: field of view = 64 mm × 64 mm, slice thickness = 2 mm, acquisition matrix = 64 × 64 pixels, in-plane resolution = 1.0 mm/pixel, number of slices = 7. The following sequence timing parameters for SEMS were used: TR = 15 s, TE = 26.5 ms, flip angle = 90°. The MRI signal within a 3.5 mm diameter region of interest was averaged over the three gradient directions and fit to a simple exponential model, using a non-linear least squares method, to obtain the ADC values.

### 4.8. Magnetic Resonance Imaging Protocol

The MRIs of the phantom with gradient temperature were performed using a standard single-slice, spin-echo sequence delivering T_2_-weighted images. Details of the MRI setup are given in the Appendix A below. The phantom was located on a dedicated cradle and placed into the quadrature birdcage resonator. The registered magnet bore temperature was 17.5 °C. Before imaging, both channels of the resonator were tuned to 128 MHz carrier frequency and matched to the 50 Ω impedance of the transmitting/receiving lines. Automatic procedures for resonant frequency and 90° pulse calibration were executed immediately prior to imaging. The details of the sequence follow: field of view = 32 × 32 mm^2^, slice thickness = 4 mm, data acquisition matrix = 32 × 32 pixels, in-plane resolution 1 mm/pixel, repetition time = 500 ms, echo time = 10.79 ms, total image acquisition time = 16 s.

The NMR data and MR images were processed using in-house developed Python platform software. The temperature map and spatial–thermal resolution were obtained using Origin (Origin 9.0, OriginLab Corporation, Northampton, MA, USA). The analysis of linear regression was conducted using Prizm software (GraphPad Prism version 5.00 for Windows, GraphPad Software, San Diego, CA, USA).

### 4.9. Biological Study

Mouse cells neuroblastoma (Neuro2a) (a kind gift from A. Bodzon-Kulakowska, Department of Biochemistry and Neurobiology, AGH University of Krakow, Poland, obtained from ATCC) were grown at 37 °C in Dulbecco’s modified Eagle medium (DMEM) supplemented with 3.7% sodium bicarbonate (NaHCO_3_), 10% fetal bovine serum and penicillin/streptomycin. All the reagents related to cell culture were obtained from Sigma-Aldrich (St. Louis, MO, USA). Cells were subcultured every 2 days until the appropriate number of cells was received for testing. After cells reached 80% confluence, they were trypsinized, seeded on sterile 96-well plates (3.0 × 10^4^ cells/cm^2^) and incubated for 24 h.

The cytotoxic activity of PEG-NPs in neuroblastoma cell lines was assessed by the MTT (3-[4,5-dimethylthiazole-2-yl]-2,5-diphenyl tetrazolium bromide) dye conversion assay. Cells (4 × 10^4^) were cultured in 0.1 mL volume of culture medium in a 96-well plate in the presence of different concentrations of PEG-NPs in obtained systems dissolved in the medium. After 24 h, the cells were washed once and further incubated for 1 h with MTT dye. The obtained blue formazan precipitate was dissolved using a solubilization buffer (5 mM HCl in isopropanol) and kept 2 h at 37 °C. The absorbance at 570 nm was measured using a microplate reader. Each result was presented as a mean of the three independent experiments, each of them performed in triplicate. SD was also calculated and presented for each mean value. The significance of the cell viability differences between no particles control and with different concentrations of MnZn ferrite nanoparticles was determined with a one-way ANOVA test with Dunnett’s test of multiple comparisons. These were conducted using Prizm software (GraphPad Prism version 5.00 for Windows, GraphPad Software, San Diego, CA, USA).

## Figures and Tables

**Table 1:** Temperature and concentration dependence of apparent coefficient (ADC) in deionized water (DIW) and agar–DIW solutions. The error in the diffusivity measurements is less than ±3%.

ADC (10^−3^ mm^2^s^−1^)				
T (°C)	DIW	0.5% Agar	1% Agar	2% Agar
5	1.34	1.33	1.29	1.27
20	2.03	2.01	1.97	1.92
37	3.00	2.97	2.91	2.83
50	3.90	3.86	3.79	3.70

**Table 2:** Comparison of main results from MnZn and CuZn studies.

Composition	Mn_0.48_Zn_0.46_Fe_2.06_O_4_	Cu_0.08_Zn_0.54_Fe_2.38_O_4_
Size: core/shell (nm)	8.5/15	6/30
M_m_ at 40 °C (Am^2^kg^−1^)	18.4	11.8
M_m_ drop in range 5–50 °C (%)	14.4	7.3
Concentration of Fe (mM/mg/mL)	0.30/0.017	2.02/0.128
T_1_ change in range 5–50 °C (%)	−16.0	−14.0
T_2_ change in range 5–50 °C	137.0	92.1
r_1_ at 40 °C (s^−1^ mM^−1^)	22.5	0.80
r_2_ at 40 °C (s^−1^ mM^−1^)	494.5	77.4
Imaging time (s)	16	256
Thermal gradient produced by:	heating by laser	cooling by Teflon block
Type of thermal gradient	radial	linear
Thermal/spatial resolution (°C/mm)	3.2/2.9	NA

**Figure A1:**
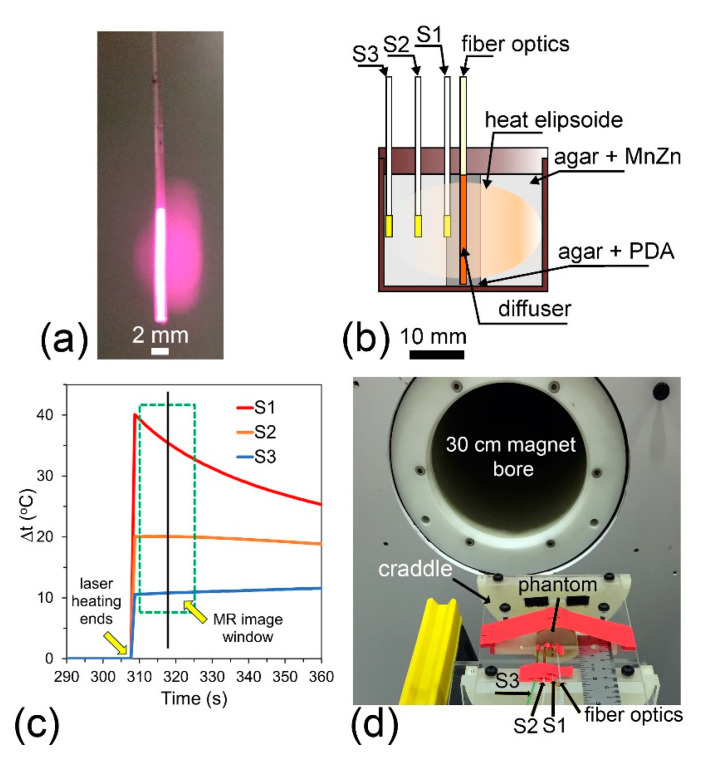
MRI setup to study the effect of temperature gradient in an agar phantom with embedded MnZn ferrite nanoparticles in concentration of 0.017 mg/mL. (**a**) Glowing light (808 nm) from the laser beam diffuser immersed in the deionized water. (**b**) The phantom with inserted optical temperature sensors (S1, S2 and S3) and the laser diffuser. (**c**) Heating curves recorded during the MRI study. Note that temperature was not recorded during laser heating due to optical sensors “blinding” by an external laser beam. The equilibrium temperature of the bulk phantom before heating started was 17.3 ± 0.4 °C. Green dashed rectangle shows 16 s MRI study interval. Black solid line points for the exact time of temperature measurement used in spatial–thermal resolution analysis. (**d**) The picture of complete setup in the front of 30 cm bore, 3.0 T magnet.

## Data Availability

The data presented in this article are available upon request to the corresponding author.

## Supplementary Materials

The supporting information can be downloaded at https://www.mdpi.com/article/10.3390/ijms242216458/s1.

## Appendix A

### Magnetic Resonance Imaging Setup

Temperature-dependent MRI experiments were conducted on the preclinical, 30 cm bore MRI scanner (Agilent, Santa Clara, CA, USA). A dedicated setup was designed and built for MRI experiments with an in-plane, radial, temperature gradient. It was based on heating the core, made of agar gel, with polydopamine with an 808 nm near-infrared diode laser (MDL-H-808-5W, CNI Optoelectronics Technology Co, Ltd., Changchun, China). PDA possesses unique properties, making photothermal conversion more efficient [58,59].

Laser light delivered through an optical fiber is dispersed sideways by a medical grade diffuser (BioTex, Inc., Houston, TX, USA) in the center of the agar–PDA core. A core, heated for 5 min, temporarily produces a strong, time-dependent, thermal gradient reaching a maximum of 30 °C over 14 mm in the surrounding jacket of the agar gel doped with MnZn ferrite nanoparticles. A diffuser is seen in action in Figure A1a dispersing light in deionized water. A schematic diagram of the phantom is shown in Figure A1b. The orange color denotes the heat ellipsoid. Profiles of heating curves recorded during the MRI study by three miniature optical sensors (S1, S2, S3) are presented in Figure A1c. The green dashed rectangle shows the time position during the 16 s imaging window. The black solid line marks the time of temperature measurement used in spatial–thermal resolution analysis. Figure A1d exhibits a picture of the complete setup assembly in front of an Agilent 30 cm preclinical scanner magnet before insertion into the isocenter of the magnet.
