# Supplementary material for: Aqueous Dispersion of Manganese–Zinc Ferrite Nanoparticles Protected by PEG as a T2 MRI Temperature Contrast Agent
Source: Int J Mol Sci. 2023 Nov 17;24(22):16458. doi: 10.3390/ijms242216458 (PMC10671015; doi:10.3390/ijms242216458)
Supplement: Supplementary file 1 [file ijms-24-16458-s001.zip › ijms-2677958-supplementary.pdf]

International Journal of
Molecular Sciences

MDPI

Supplementary Information

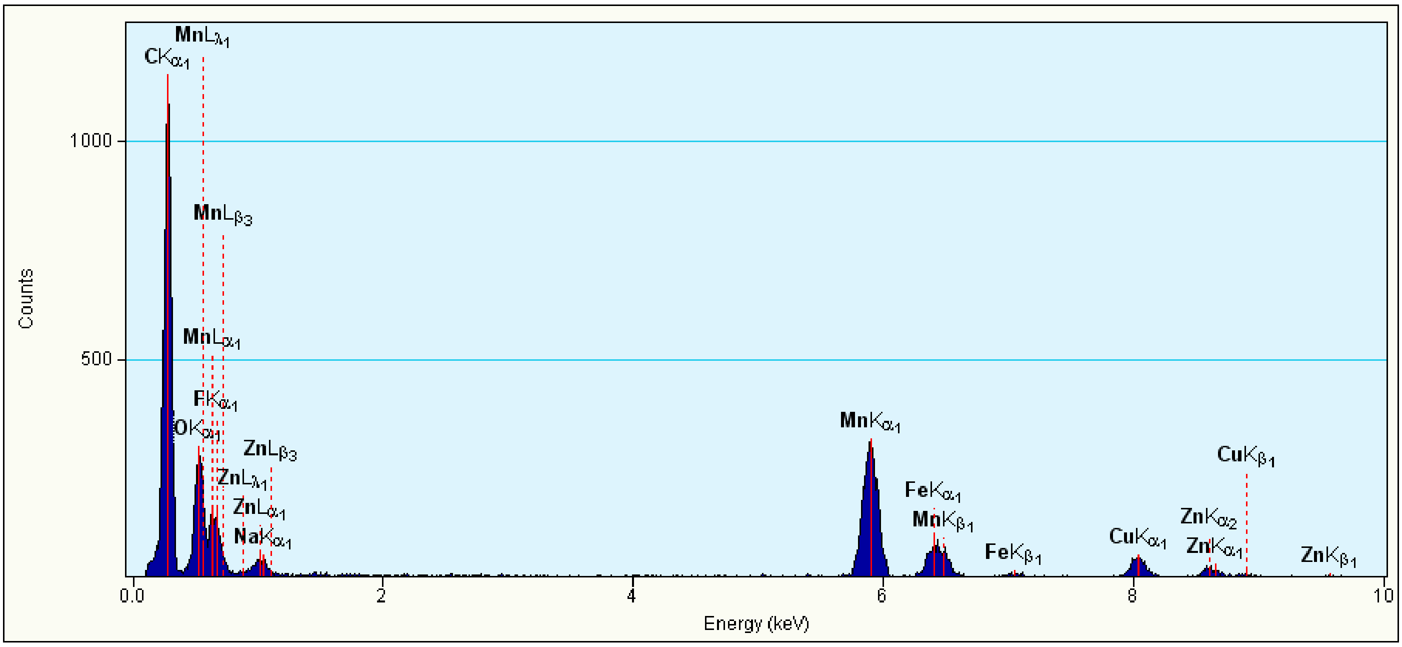

Figure S1. EDX spectrum of obtained nanoparticles

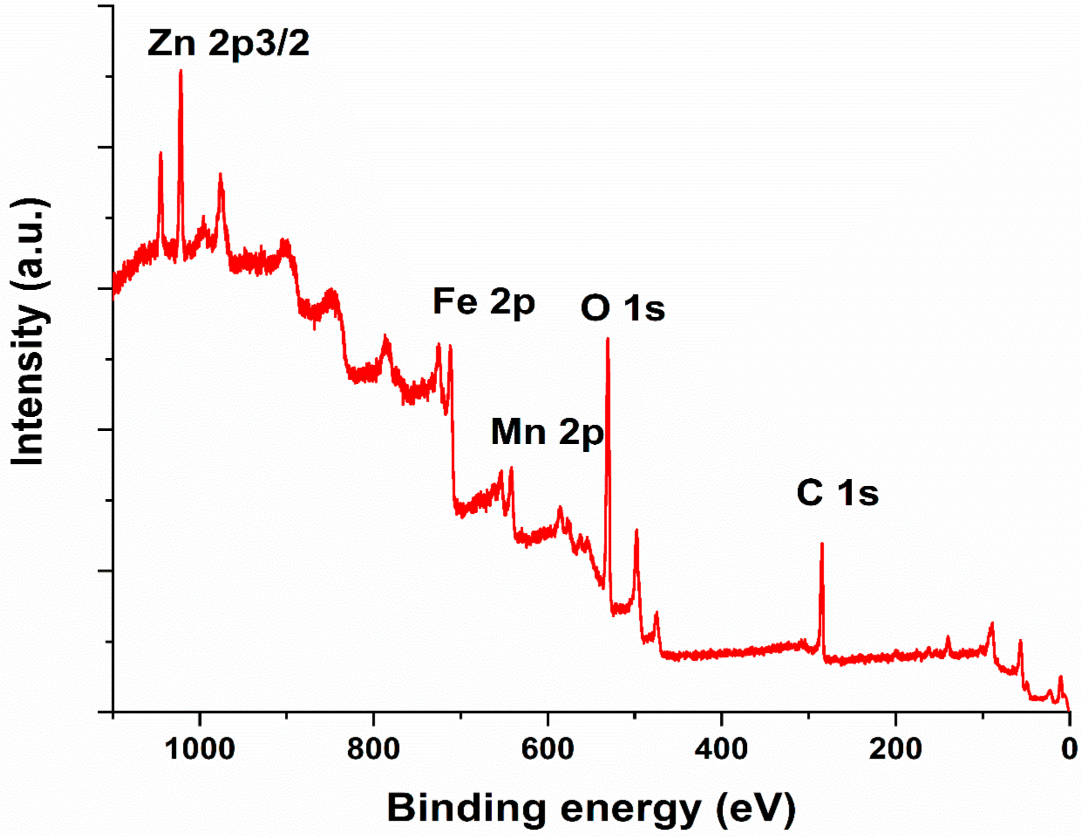

Figure S2. XPS survey spectrum of obtained nanoparticles

Table S1 . Atomic concentration table of the XPS measurement.

Electron shell	*Amount[%at]*
C 1s	36.20
O 1s	40.19
Mn 2p3	5.35
Fe 2p3	13.29
Zn 2p3	4.97

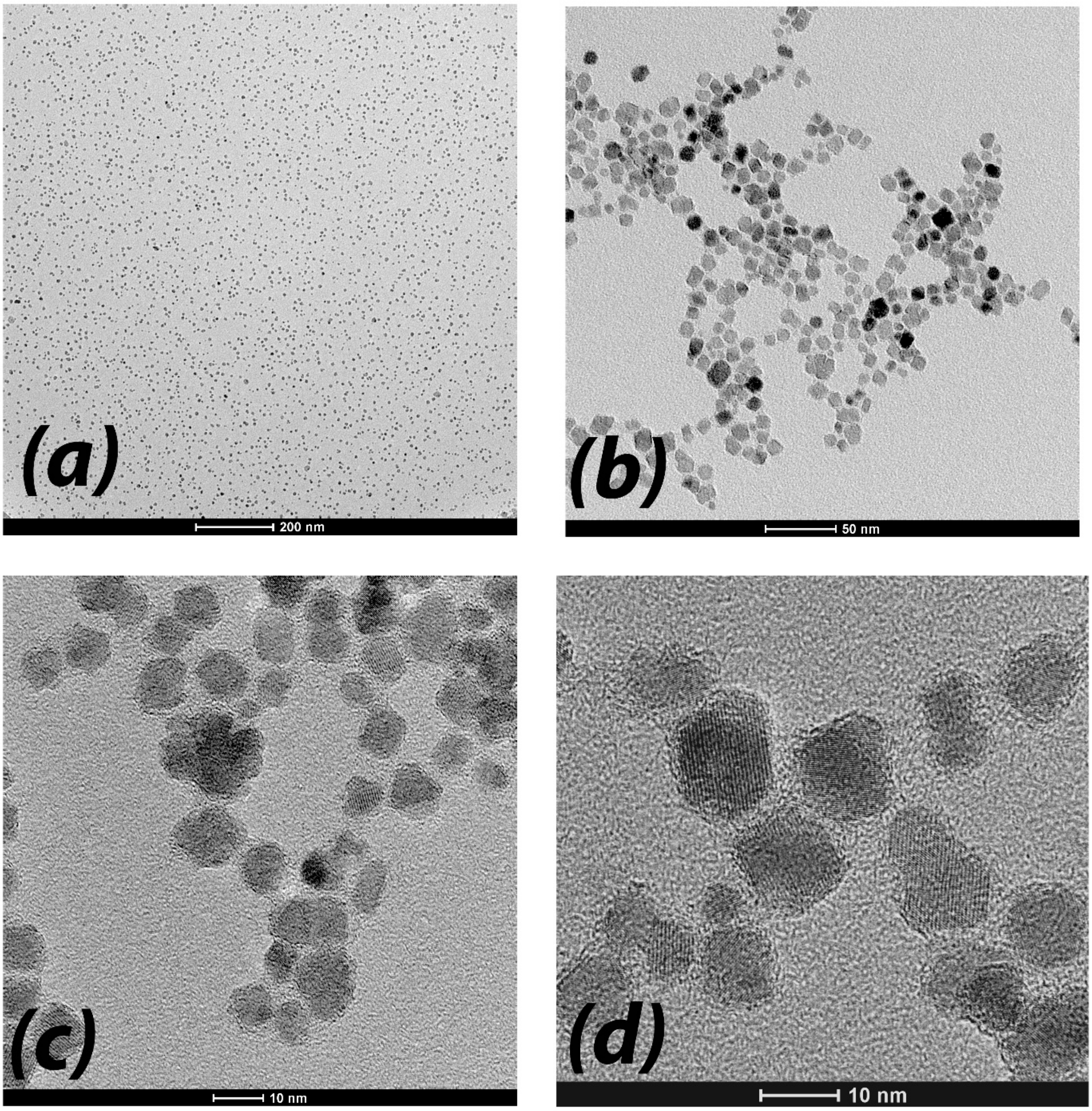

Figure S3. a,b TEM image and c,d high-resolution TEM (HRTEM) image of the obtained nanoparticles

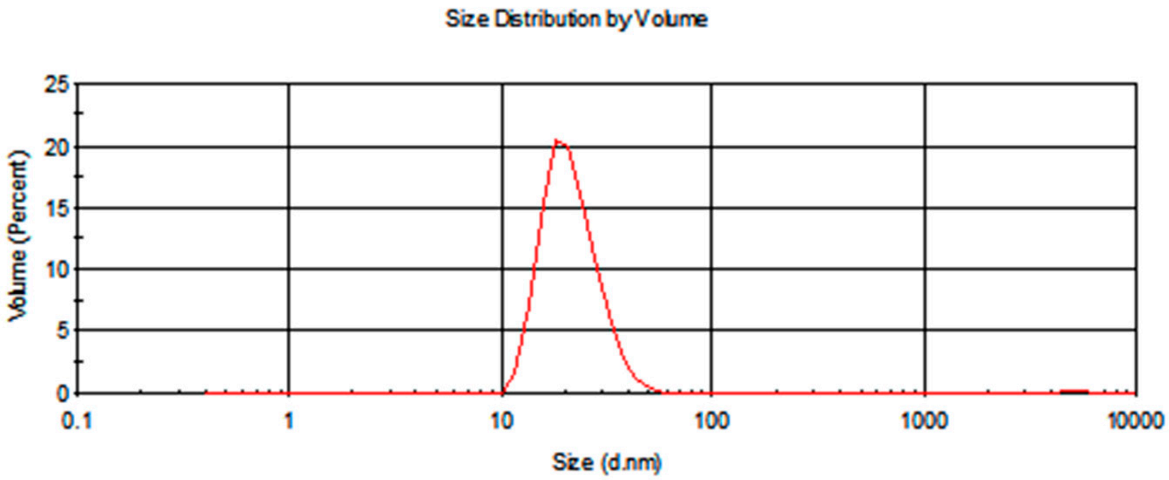

Figure S4. Size distribution analysis by dynamic light scattering (DLS). Nanoparticles were dispersed in deionized water
